# Application of O-RADS Ultrasound Lexicon-Based Logistic Regression Analysis Model in the Diagnosis of Solid Component-Containing Ovarian Malignancies

**DOI:** 10.1155/2022/7187334

**Published:** 2022-10-25

**Authors:** Hui Luo, Ziqing Lin, Lijuan Wu, Yuying Wang, Haojie Ning, Yanping Feng, Yulu Cheng, Xiaoyi Wen, Xiaoyan Liu

**Affiliations:** ^1^Department of Ultrasound, South Medical University Affiliated Maternal & Child Health Hospital of Foshan, Foshan 528000, China; ^2^Department of Pathology, South Medical University Affiliated Maternal & Child Health Hospital of Foshan, Foshan 528000, China

## Abstract

**Objective:**

To use the logistic regression model to evaluate the value of ultrasound characteristics in the Ovarian-Adnexal Reporting and Data System ultrasound lexicon in determining ovarian solid component-containing mass benignancy/malignancy.

**Methods:**

We retrospectively analyzed the data of 172 patients with adnexal masses discovered by ultrasound, and diagnosis was confirmed by postoperative pathological tests from January 2019 to December 2021. Thirteen ovarian tumor-related parameters in the benign and malignant ovarian tumor groups were selected for univariate analyses. Statistically significant parameters were included in multivariate logistic regression analyses to construct a logistic regression diagnosis model, and the diagnostic performance of the model in predicting ovarian malignancies was calculated.

**Results:**

Of the 172 adnexal tumors, 104 were benign, and 68 were malignant. There were differences in cancer antigen 125, maximum mass diameter, maximum solid component diameter, multilocular cyst with solid component, external contour, whether acoustic shadows were present in the solid component, number of papillae, vascularity, presence/absence of ascites, and presence/absence of peritoneal thickening or nodules between the benign ovarian tumor and malignancy groups (*p* < 0.05). Logistic regression analyses showed that maximum solid component diameter, whether acoustic shadows were present in the solid component, number of papillae, and presence/absence of ascites were included in the logistic regression model, and the area under the receiver operating characteristic curve for this regression model in predicting ovarian malignancy was 0.962 (95% confidence interval: 0.933~0.990; *p* < 0.001). Logit (*p*) ≥ −0.02 was used as the cutoff value, and the prediction accuracy, sensitivity, specificity, positive predictive value, and negative predictive values were 93.6%, 86.8%, 98.1%, 96.7%, and 91.9%, respectively.

**Conclusion:**

The logistic regression model containing the maximum solid component diameter, whether acoustic shadows were present in the solid component, number of papillae, and presence/absence of ascites can help in determining the benignancy/malignancy of solid component-containing masses.

## 1. Introduction

The mortality rate of ovarian cancer is the highest among gynecological cancers. The latest study reported that there were 313,959 new cases of ovarian cancer globally in 2020 and 207,252 deaths. Hence, ovarian cancer is an important disease that threatens women's health [1, 2]. To eliminate ambiguities in ultrasound reports and improve the accuracy of preoperative benignancy/malignancy risk assessment for ovarian masses, the American College of Radiology (ACR) Ultrasound Working Group published the Ovarian-Adnexal Reporting and Data System (O-RADS) ultrasound risk stratification and management consensus guidelines [3] in 2020. The ultrasound lexicon in the guidelines includes lesion descriptions, definitions, explanations, and malignancy risk classification of masses. In this study, the O-RADS ultrasound lexicon was used as a basis and combined with patient age, menopause status, and cancer antigen 125 (CA125) test to construct a logistic regression model, and the value of various variables in determining the benignancy/malignancy of solid component-containing masses was assessed.

## 2. Data and Methods

### 2.1. Study Subjects

We retrospectively analyzed the data of patients who underwent surgery due to the discovery of adnexal masses by ultrasound from January 2019 to December 2021. Patients underwent preoperative CA125 test, and ultrasound examination was performed 1 week before surgery. The ultrasound images were clear, and diagnosis report descriptions were complete. There were clear postoperative pathological results. After excluding patients with typical benign lesions, unilocular cysts, and multilocular cysts defined in the O-RADS ultrasound lexicon, a total of 172 patients with adnexal masses containing solid components were enrolled. The ages of patients ranged from 15 to 85 years, and the mean age was 40.05 ± 13.11 years. This study was approved by the ethics committee of the hospital.

### 2.2. Instruments and Equipment

The ultrasound equipment used were Samsung UGEO WS80A, Canon Aplio300, and Mindray Resona8 Elite color Doppler ultrasound diagnostic devices equipped with an abdominal probe and intraluminal probe. The intraluminal probe frequency was 3–10 MHz, and the abdominal probe frequency was 1–5 MHz. Patient images were viewed on the Picture Archiving and Communication System imaging system. The Alinity i system fully automated analyzer (Abbott Laboratories, Chicago, IL, USA) was used to measure serum CA125 levels.

### 2.3. Data Analyses

Two experienced ultrasound physicians (working in gynecological ultrasound examination for more than 10 years), who were blinded to the pathological results, conducted classification and interpretation of image features. When the imaging results were different, the physicians discussed until a consensus was reached before recording the interpretation. Thirteen parameters, namely, age, hysterectomy or not, menopause status, CA125, mass characteristics, maximum mass diameter, maximum solid component diameter, external contour, whether acoustic shadows were present in the solid component, number of papillae, vascularity signal grade, presence/absence of ascites, and presence/absence of peritoneal thickening or nodules of all patients, were recorded. Menopause was defined as the absence of menstruation for more than 12 months.

### 2.4. O-RADS Ultrasound Lexicon-Based Image Classification Criteria

The mass characteristics were as follows. (1) Solid unilocular mass: the unilocular cyst contains a measurable solid component or at least one papillary projection, and the maximum diameter of the solid component or papillary projection is ≥3 mm; (2) solid multilocular mass: multilocular cysts with measurable solid component or at least one papillary projection and the maximum diameter of the solid component or papillary projection are ≥3 mm; and (3) solid mass: two-dimensional grayscale ultrasound showed that the solid component in the mass accounted for 80% of the tumor and could include a papillary projection or small cysts protruding into the mass. External contours were smooth (regular outer margin) or irregular (nonuniform outer margin), and a lobulated outer margin was considered irregular. Acoustic shadows are artifacts caused by acoustic attenuation after tissues absorb sound waves. Papillae are solid components with cystic wall or high septa ≥ 3 mm that protrude into the cyst and should be counted as the number of papillae. Ascites refers to fluid extending above the uterine fundus beyond the pouch of the Douglas or cul-de-sac when anteverted/anteflexed and anterior/superior to uterus when retroverted/retroflexed. Peritoneal thickening or nodules are nodularity or diffuse thickening of the peritoneal lining(s) or along the bowel serosal surface or peritoneum associated with peritoneal carcinomatosis. Vascularity is as follows: color scores 1–4, overall assessment of color Doppler flow within the entire lesion: 1-no flow, 2-minimal flow, 3-moderate flow, and 4-very strong flow.

### 2.5. Statistical Analyses

SPSS20.0 statistical software was used for the statistical analyses. Age was quantitative data, expressed as $\overset{¯}{x}\pm s$, and the independent samples *t*-test was used for intergroup comparison. The mean maximum diameter is expressed as the median (*M*), and Mann–Whitney *U* test was used for intergroup comparisons. Qualitative data are expressed as the number of patients, and the *χ*^2^ test was used for univariate analyses. Pathological results were used as a dependent variable to construct the logistic regression diagnosis model. The corresponding regression coefficient (*β*), standard error (SE), and OC (95% confidence interval [95% CI]) were obtained. The receiver operating characteristic (ROC) curve of the logistic regression model in ovarian malignancy diagnosis was plotted, and its diagnostic performance was analyzed. *p* < 0.05 was considered statistically significant.

## 3. Results

### 3.1. Histopathological Category Distribution of Ovarian Tumors

Of the 172 adnexal tumors, postoperative histopathology tests showed that 104 were benign and 68 were malignant. Of the benign tumors, 29 were fibrothecomas, 28 were benign teratomas, 14 were mucinous and serous cystadenomas, 12 were serous and mucinous adenofibromas, 7 were fallopian tube effusion or empyema, 6 were ovarian endometriomas, 4 were simple cysts, 2 were fallopian tube tuberculosis, and 2 were Brenner tumors. Of the malignant tumors, 30 were serous, mucinous, and endometrioid borderline tumors, 9 were serous and mucinous cystoadenocarcinomas, 8 were endometrioid adenocarcinomas, 6 were clear cell carcinomas, 5 were adult granulosa cell tumors, 3 were metastases, 3 were mixed germ cell tumors, and 1 patient each had stromal carcinoid of ovary, yolk sac tumor, immature teratoma, and moderately differentiated Sertoli-Leydig cell tumor.

### 3.2. Intergroup Comparison of Factors in the Benign and Malignant Groups

**Figure 1:**
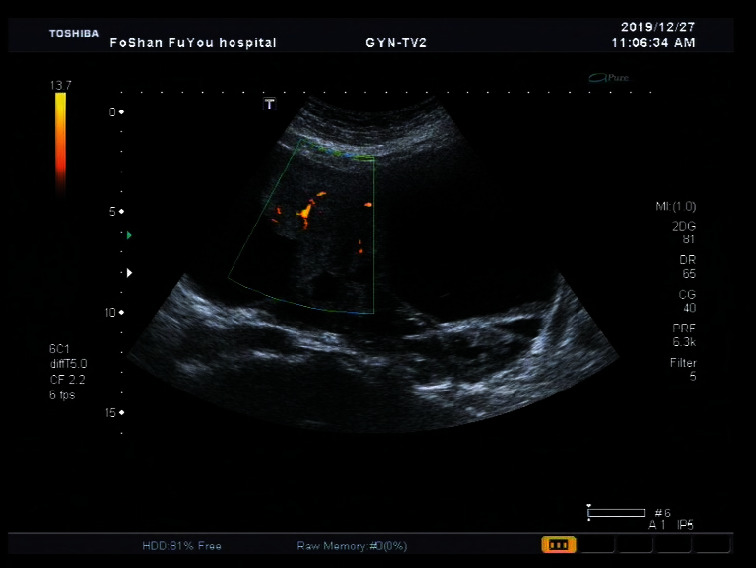
Large multilocular solid mass in pelvic cavity, maximum diameter 196 mm, and maximum diameter of solid component about 112 mm. The pathological result was endometrioid adenocarcinoma.

**Figure 2:**
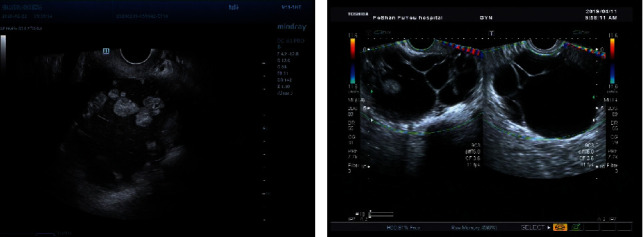
Both images show multilocular cyst with solid component, >3 papillary echoes could be seen in the mass on the right, and the pathological result was borderline serous tumor. There are less than 3 papillae on the right, and the pathological result was serous papillary adenofibroma.

**Figure 3:**
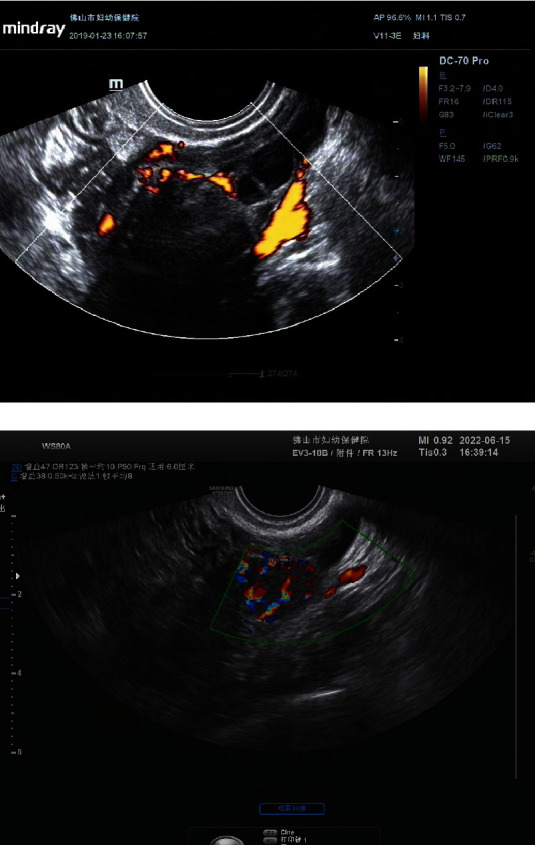
Both images show solid with hypoechoic. The maximum diameter of the mass on (a) was about 33 mm, with acoustic shadow and color score of 3, and the pathological result was ovarian fibrothecoma. The maximum diameter of the mass on (b) was about 20 mm, acoustic shadow absent, color score of 4, ascites were seen, and the pathological result was adult granulosa cell tumor.

**Figure 4:**
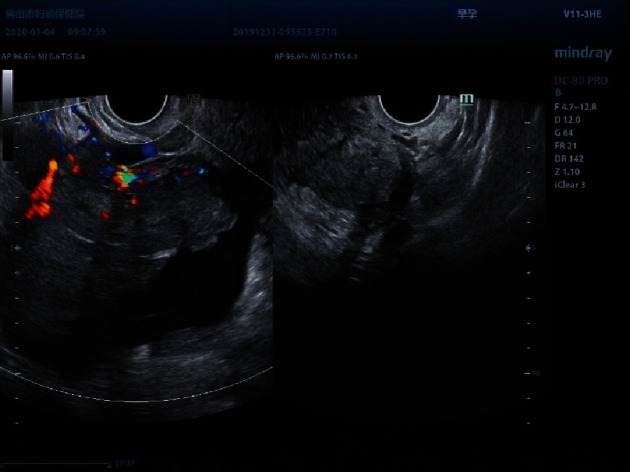
A solid mass with a maximum diameter of 158 mm in the right adnexa, irregular external contour, color score of 3, ascites, and peritoneal thickening were seen. The pathological result was high-grade serous cystadenocarcinoma.

Comparison of the benign and malignant groups showed that there were differences in CA125, maximum diameter, maximum solid component diameter, multilocular cyst with solid component, external contour, whether acoustic shadows were present in the solid component, number of papillae, vascularity, presence/absence of ascites, and presence/absence of peritoneal thickening or nodules between the benign ovarian tumor and malignancy groups, and these differences were statistically significant (all *p* < 0.05, Table 1; Figures 1–4).

### 3.3. Logistic Regression Analysis Results of Correlation between Various Factors and Ovarian Malignancies

Pathological results were used as the dependent variable, and statistically significant feature values in the univariate analyses (Table 2) were used for bivariate stepwise logistic regression analyses. From the results, four statistically significant independent variables (maximum solid component diameter, whether acoustic shadows were present in the solid component, number of papillae, and presence/absence of ascites) were included in the logistic regression model (all *p* < 0.05; Table 3).

### 3.4. ROC Curve of the Logistic Regression Model Predicts Ovarian Tumor Benignancy/Malignancy

**Figure 5:**
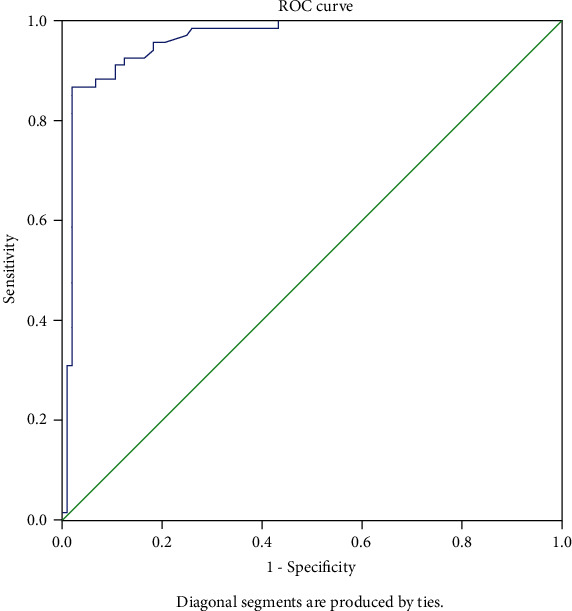
Receiver operating characteristic curve of the regression model in diagnosing malignant ovarian tumors.

A logistic regression model to predict ovarian tumor benignancy/malignancy was constructed, Logit (*p*) = −2.261 + 0.037^∗^X3 − 5.318^∗^X6 + 4.280^∗^X7 + 3.678^∗^X9. The area under the ROC curve for this regression model in predicting ovarian malignancy was 0.962 (95% confidence interval: 0.933~0.990; *p* < 0.001). Logit (*p*) ≥ −0.02 was used as the cutoff value and the prediction accuracy, sensitivity, specificity, positive predictive value, and negative predictive value were 93.6%, 86.8%, 98.1%, 96.7%, and 91.9%, respectively (Figure 5).

## 4. Discussion

The ACR recommends transvaginal/transabdominal ultrasound as the preferred radiologic examination method for ovary-adnexal masses [4]. However, the histopathology of ovarian tumor tissues is complex, and there are many differences in ultrasound image features. Currently, there is no unified diagnostic criteria, and there is a strong reliance on the operator. Hence, there is some difficulty in determining tumor benignancy/malignancy [5]. To increase the preoperative diagnostic accuracy of ovarian tumors, many ultrasound-based integrated scoring systems have been proposed and used, such as Risk of Malignancy Index, gynecologic imaging-RADS, International Ovarian Tumor Analysis logistic regression models, simplified rules, and ADNEX mode [6–9]. The O-RADS ultrasound reporting and data system is the only classification system with a lexicon at present and includes all risk categories and related management protocols. O-RADS has high sensitivity for determining the benignancy/malignancy of appendage masses [10]. However, the malignancy rate of O-RADS 4 category lesions in ACR is large (10–50%), and there is still a possibility of misdiagnosis and missed diagnosis when relying on routine color Doppler ultrasound. As this paper was based on the O-RADS ultrasound lexicon, we selected 13 factors affecting ovarian tumor benignancy/malignancy and constructed a regression model to evaluate the value of each factor in determining the benignancy/malignancy of solid component-containing ovarian masses.

**Figure 6:**
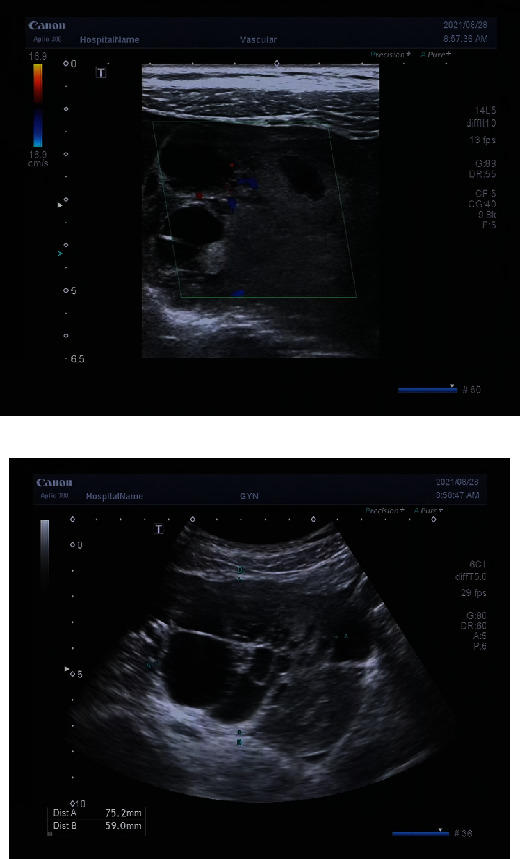
The patient was 14 years old. A multilocular solid mass was seen in the right appendage, the acoustic shadow was absent in the posterior solid component, mild ascites was seen, and the pathological result was malignant germ cell tumor.

The malignancy rate of solid component-containing ovarian masses is higher than simple cystic masses, and this is also a challenge in determining ovarian tumor benignancy/malignancy. A study carried out subclassification of multilocular cysts, solid lesions, and other cystic lesions with solid components as O-RADS 4 lesions and malignancy rates were 17.02% and 42.57% [11], respectively. This shows that the solid component has a greater impact on tumor benignancy/malignancy. The O-RADS ultrasound lexicon clearly defined the solid component. In this study, we excluded typical benign lesions and included 172 ovarian masses containing solid component. A study found that the incidence of ovarian malignancy increased with age but the difference was not significant [12]. This study compared the 13 influencing factors of benign/malignant ovarian tumor and found that there were no statistically significant differences in age and menopause status between the two groups, which was similar to the results of Li et al. [13]. This may be due to the complex histopathology of ovarian tumors, and different pathological types of ovarian cancer have different pathogenesis and biological behaviors. For example, epithelial ovarian cancer mostly occurs in postmenopausal women, and malignant germ cell tumor tends to occur in children and women undergoing puberty. In this study, one patient with yolk sac tumor was 15 years old, and one patient with malignant mixed germ cell tumor was only 14 years old (Figure 6), which may have been due to the low sample size and selection bias.

In this study, logistic regression analyses were used to identify the following four independent risk factors predicting ovarian malignancy by screening: maximum solid component diameter, whether acoustic shadows were present in the solid component, number of papillae, and presence/absence of ascites. The results showed that of the 63 masses with acoustic shadows, only 2 were confirmed to be malignant ovarian tumor after surgery. Hence, acoustic shadows may be a critical characteristic for ruling out malignancy, consistent with the results of Cao et al. [11]. However, acoustic shadow is not included in the O-RADS risk stratification system, which may lead to overclassification of fibrothecomas, broad ligament fibroids, and other benign masses with high fiber content and affect the diagnostic accuracy of O-RADS4 and five categories. However, acoustic shadows have been considered a potential revision factor for future O-RADS [14]. In this study, a logistic regression model to predict ovarian tumor benignancy/malignancy was constructed. The area under the ROC curve for this regression model in predicting ovarian malignancy was 0.962 (95% CI: 0.933~0.990; *p* < 0.001), logit (*p*) ≥ −0.02 was used as the cutoff value, and the prediction accuracy, sensitivity, specificity, positive predictive value, and negative predictive value were 93.6%, 86.8%, 98.1%, 96.7%, and 91.9%, respectively, demonstrating good diagnostic performance. Thus, the four included factors may help in O-RADS4 category subclassification.

One limitation of this study is that it was a retrospective analysis, which may have incomplete image information and possibly some bias due to the low sample size. A large prospective study should be carried out in the future to examine the correlation between O-RADS 4 category subclassification and menopause age and malignant ovarian tumor.

In summary, the logistic regression model containing the maximum solid component diameter, whether acoustic shadows were present in the solid component, number of papillae, and presence/absence of ascites can help in determining the benignancy/malignancy of solid component-containing masses, and acoustic shadow is a critical characteristic for excluding malignant tumors.

## Figures and Tables

**Table 1:** Univariate analyses of ovarian tumor benignancy/malignancy.

	Benign (*n* = 104)	Malignant (*n* = 68)	*t/Z/χ* ^2^	*p*
Age (years)	40.40 ± 13.45	39.50 ± 12.64	*t* = 0.441	0.660
CA125 (U/ml)	17.20 (11.60, 32.90)	93.30 (26.45, 420.50)	*Z* = −5.978	0.001
Maximum diameter (mm)	52.00 (39.25, 79.75)	87.00 (63.50, 144.00)	*t* = −5.898	0.001
Maximum diameter of solid component (mm)	30.00 (20.00, 41.75)	45.50 (25.75, 80.25)	*Z* = −4.275	0.001
Menopause status				
No	72 (62.9)	50 (73.5)	*χ* ^2^ = 0.368	0.544
Yes	32 (30.8)	18 (26.5)		
Hysterectomy or not				
No	101 (97.1)	67 (98.5)	*χ* ^2^ = 0.362	0.547
Yes	3 (2.9)	1 (1.5)		
Unilocular cyst with solid component				
No	64 (61.5)	45 (66.2)	*χ* ^2^ = 0.381	0.537
Yes	40 (38.5)	23 (33.8)		
Multilocular cyst with solid component				
No	84 (80.8)	46 (67.6)	*χ* ^2^ = 3.836	0.049
Yes	20 (19.2)	22 (32.4)		
Solid				
No	60 (57.7)	45 (66.2)	*χ* ^2^ = 1.245	0.265
Yes	44 (42.3)	23 (33.8)		
External contour				
Irregular	2 (1.9)	20 (29.4)	*χ* ^2^ = 27.853	0.001
Smooth	102 (98.1)	48 (70.6)		
Acoustic shadow in solid component				
Absent	43 (41.3)	66 (97.1)	*χ* ^2^ = 54.981	0.001
Present	61 (58.7)	2 (2.9)		
Number of papillae				
0–3	101 (97.1)	37 (54.4)	*χ* ^2^ = 47.276	0.001
>3	3 (2.9)	31 (45.6)		
Vascularity				
1	62 (59.6)	3 (4.4)	*χ* ^2^ = 71.632	0.001
2	39 (37.5)	35 (51.5)		
3	2 (1.9)	25 (36.8)		
4	1 (1.0)	5 (7.4)		
Ascites				
Absent	101 (97.1)	43 (63.2)	*χ* ^2^ = 34.629	0.001
Present	3 (2.9)	25 (36.8)		
Peritoneal thickening or nodules				
Absent	103 (99.0)	60 (88.2)	*χ* ^2^ = 9.677	0.001
Present	1 (1.0)	8 (11.8)		

**Table 2:** List of assigned values for various factors in logistic regression of ovarian malignancy correlation.

	Assigned value
*X*1 CA125 U/ml	Continuous variable
X2 maximum diameter	Continuous variable
X3 maximum diameter of solid component	Continuous variable
X4 multilocular cyst with solid component	1 = present, 0 = absent
X5 external contour	1 = smooth, 0 = irregular
X6 presence of acoustic shadow in solid component	1 = absent, 0 = present
X7 number of papillae	1 = ^“^ > 3^”^, 0 = ^“^0–3^”^
X8 vascularity	1 = 1, 2 = 2, 3 = 3, 4 = 4
X9 presence of ascites	1 = present, 0 = absent
X10 peritoneal thickening or nodules	1 = present, 0 = absent

**Table 3:** Logistic regression analysis results of ovarian malignancy correlation.

	*β*	SE	Wald	*p*	OC	OC 95% CI	
						Lower limit	Upper limit
X3 maximum diameter of solid component	0.037	0.013	7.711	0.005	1.038	1.011	1.065
X6 presence of acoustic shadow in solid component	-5.318	1.900	7.830	0.005	0.005	0.000	0.203
X7 number of papillae	4.280	1.138	14.147	0.001	72.249	7.766	672.150
X9 presence of ascites	3.678	1.404	6.865	0.009	39.584	2.526	620.234

## Data Availability

The data that support the findings of this study are available from the corresponding author upon reasonable request.
